# All in a scent - naphthalene dependence confined to pregnancy: a case report

**DOI:** 10.1186/s12888-022-04369-1

**Published:** 2022-11-19

**Authors:** Isuri Wimalasiri, Chathurie Suraweera

**Affiliations:** University Psychiatry Unit, National Hospital of Sri Lanka, Colombo, Sri Lanka

**Keywords:** Naphthalene dependence, Moth balls, Pregnancy

## Abstract

**Background:**

Inhalant or volatile substance use is a health issue with significant medical and psychiatric sequelae. Inhalants or volatile substances are volatile organic substances found in domestic and commercial products which are inhaled to obtain pleasurable effects. They are easily accessible, cheap, and legal. Common inhalants are spray, paints, glue and shoe polish whilst naphthalene or ‘mothball’ abuse is reported less commonly. We report a case of a 29-year-old female who inhaled and ingested naphthalene during her pregnancy. This case report is unique because the dependence on naphthalene was confined to pregnancy and resolved as soon as she delivered. This brings up the question whether pregnancy in general increases the risk of substance dependence in vulnerable populations or whether the dependence in this patient during pregnancy is due to individual factors.

**Case presentation:**

The patient we report is a 29-year-old female who developed a strong desire to inhale mothballs during her third pregnancy. The pattern of use started in the first trimester meeting the criteria for dependence syndrome and resolved completely by the second day following delivery. She had features suggestive of harmful use in her second pregnancy as well.

**Conclusions:**

The case report emphasizes that pregnant women should be screened for psychoactive substance use. Equally important is the need for adequate psychoeducation about the myths and cultural beliefs associated with pregnancy-related cravings and the potentially devastating consequences of harmful cravings on the neonate and the mother. The case highlights how chemicals used in day-to-day activities can lead to dependence.

## Background

Psychoactive drugs are substances that, when taken in or administered into one’s system, affect mental processes including, perception, consciousness, cognition, mood and emotions [1]. Use of inhalants, also known as volatile substances or solvents, is the deliberate inhalation of such substances to achieve an altered mental state [2]. Sniffing, huffing and bagging are terms associated with inhalant use; it is a health issue with significant medical and psychiatric sequelae.

Inhalants or volatile substances are organic substances found in domestic and commercial products. They are easily accessible, cheap, and legal. Common inhalants are glue, shoe polish, toluene, spray paints, gasoline, and lighter fluid whilst naphthalene or ‘mothball’ use is reported less commonly [3]. The pharmacological action of inhalants in the central nervous system is not clearly defined, although it is postulated to increase the fluidity of the neuronal cell membranes. Additionally, inhalants may exert their action via many ion channels resulting in increased Gamma-aminobutyric acid (GABA) function and decreased N-methyl-D-aspartate (NMDA) receptor activity [4].

Mothballs contain two main chemicals, namely naphthalene and paradichlorobenzene [5]. Naphthalene is the primary chemical substance in mothballs and is a volatile chemical that is misused as an inhalant. There are only a few published case reports on the use of naphthalene [6, 7]. Understandably, the incidence and prevalence of naphthalene use are difficult to estimate. However, as is the case with other inhalant and volatile substance use, the rates are presumably higher among the teenage and young adult population [4]. The inhalation of naphthalene produces a rapid ‘high’, manifested as euphoria and generalized intoxication. Chronic use can be associated with impairment of vital organs like the liver, and cardiac dysrhythmias and deranged end-tidal gas are observed rarely. Prolonged exposure can result in hepatic failure and severe hemolytic anaemia.

We report a case of a 29-year-old female who inhaled and ingested naphthalene during her pregnancy. The case report is unique because the dependence on naphthalene was limited to pregnancy and resolved as soon as she delivered. The case report highlights the need for the clinicians to be aware of rare and uncommon presentations of dependence on inhalants which otherwise may lead to adverse effects.

## Case presentation

The patient we report is a 29-year-old Sri Lankan female who is a mother of three, a housewife from a suburban area in Sri Lanka. She was referred to psychiatric services by the maternity hospital, where she was admitted for delivery of her third child. She divulged the fact that she has been sniffing and ingesting mothballs to the intern house-officer who was taking history related to her dietary habits. Although the patient mentioned this fact nonchalantly, the house-officer had immediately noted the importance of it and decided to refer her to psychiatric services. This was her first contact with psychiatric services, and we assessed her on the third day following delivery.

Our patient was referred to psychiatric services as she had a strong desire to smell mothballs (naphthalene) which lasted throughout her pregnancy. She developed a desire to smell mothballs in the early part of the first trimester of current pregnancy. The desire progressively worsened as the pregnancy advanced with buying mothballs and keeping approximately 10 to 20 mothballs in a cloth bag, which she smelled and carried throughout the day. By the third trimester, she started grinding mothballs and inhaling the powder to obtain a stronger smell.

A few weeks before delivery, she had begun to ingest a pinch of powdered mothballs once or twice a day. However, she had not developed any physical symptoms of naphthalene intoxication following ingestion. The patient continued to inhale and ingest mothballs despite knowing that they may be harmful to her and she became irritable when she had no access to mothballs. She was verbally aggressive towards her husband and two older children. She had no control over the inhalation of mothballs, particularly during the third trimester. On a typical day, she would start smelling the mothball bag while having morning tea and repeating inhalation three to four times a day, each episode lasting about ten to fifteen minutes.

She was admitted to the maternity hospital for delivery at term. Her baby was delivered by normal vaginal delivery, weighed 2.8 kg and was healthy. The baby did not show any withdrawal or toxicity features and there were not any NICU admissions. Lactation was established well, and our patient did not have difficulty in bonding with her baby or any postpartum complications. She did not have access to her mothballs after admission to the maternity hospital. The last mothball inhalation was four days before the first assessment, the day before delivery. Our patient reported that her craving for mothballs resolved the day after delivery, and she was confident that she would not use naphthalene again following delivery.

The patient revealed that she developed a similar craving for mothballs in the latter part of her second pregnancy. However, this has been occasional, and she did not resort to ingesting mothballs. Identical to the current episode, her craving for mothballs had entirely resolved following delivery. She had not used alcohol or any other psychoactive substances, and she did not have any depressive, manic, or psychotic episodes in the past. She did not have pica or any other abnormal eating habits in the past. She had never engaged in self-harm or contemplated suicide.

There was no family history of substance use or mental illness. Our patient was educated up to General Certificate of Education (G.C.E.), ordinary level examination and did not have any evidence of a learning disability. She could attend to household activities, caring for her two older children, aged five and three years. Her two older daughters were healthy, and she received good support from her husband, a boutique owner. Her medical history was unremarkable, and she did not have any nutritional deficiencies. Her haemoglobin level had been within the normal range for a pregnant woman throughout the pregnancy. Her premorbid personality did not suggest any abnormal personality traits, and her coping strategies included venting her emotions to a friend or a relative.

On physical and mental status examination, she was a petite female dressed in maternity clothes and willingly engaged in conversation. She did not have any tremors or features suggestive of a physiological withdrawal syndrome. It was easy to build up rapport with her. Her speech was normal in rate, volume, and amount, and it was coherent and rational. She was euthymic and did not have any thought or perceptual abnormality. Her cognitive functions were intact, with no evidence of a learning disability. The patient had good insight regarding her naphthalene dependence and was in the contemplative stage with regards to abstinence. The physical examination of the patient was unremarkable with no signs of naphthalene toxicity.

## Management Plan

Based on the above features the diagnosis of naphthalene dependence was made. The management of the patient we reported included explaining the harmful effects of her behaviour, assessing her motivation to abstain from naphthalene inhalation and ingestion, and liaising with the obstetric and neonatology teams to monitor the newborn for any side effects. We trained her on how to control her craving to prevent future relapses. The multidisciplinary team was employed in her management; the social worker contacted her husband to obtain collateral history. The collateral history from her husband revealed that the patient is very caring towards the children. There was unlikely to be any dispute about her parenting capacity. Her husband also knew about her habit of mothball sniffing however did not think it would be harmful to her or the baby. She was discharged three days after the delivery. We continued to follow her up after the discharge.

During follow-up sessions, she remained abstinent, and her baby was thriving well. Three months following delivery, she was functioning well and was planning for permanent sterilization. With no foreseeable future pregnancies, our patient believed that she would not inhale or consume naphthalene in the future.

## Discussion and conclusion

The patient we report met the International Classification of Diseases (I.C.D.)-10 criteria for the dependence on naphthalene. Out of the six ICD- 10 criteria for substance use, she met at least five criteria including, a strong desire or a sense of compulsion for the substance, difficulty in controlling substance taking behaviour, tolerance, progressive neglect of alternative pleasures and continued use despite knowing harmful effects of use [8]. All of these symptoms have been present within the last year, hence, she qualifies for a diagnosis of mental and behavioural disorders due to use of volatile solvents, dependence syndrome (F18.2). However, it was clear that substance use was limited to pregnancy as the use commenced in the first trimester and resolved the day after delivery. This pattern was also observed during her second pregnancy to a lesser degree. Therefore, her decision to undergo a permanent sterilization method would be a protective factor against future risk of substance use since naphthalene dependence recurred only during pregnancies.

Available literature reveals adverse effects of chronic inhalation of mothballs, including deranged liver function tests, haemolytic anaemia, and chronic renal failure [9]. Ingestion of mothballs can lead to haemolytic anaemia, hyperbilirubinaemia, and acute kidney injury. Some case reports reveal death due to mothball ingestion, and a conspicuous feature is a delay between the ingestion and the onset of symptoms [10].

Naphthalene and its metabolic products cross the placenta. There are case reports of methaemoglobinaemia and haemolytic anaemia of the newborn associated with naphthalene inhalation by the mother during pregnancy. Other symptoms of perinatal naphthalene toxicity after maternal ingestion of mothballs in pregnancy include hypotension, hyperbilirubinaemia, sepsis and pulmonary hypertension [6, 7]. Fortunately, our patient’s baby was healthy according to the assessment by the neonatology team. It is worth emphasizing the need for a thorough maternal history. Unlike the patient we reported, who willingly revealed her naphthalene use to the obstetric team, some patients might not reveal their use. The baby might clinically deteriorate rapidly if not treated promptly [6].

Furthermore, neonates are more susceptible to naphthalene poisoning as they have thinner skin and lower glutathione levels, making them more vulnerable to oxidative stress. If the mother frequently handles naphthalene, the baby might develop features of toxicity even by skin contamination [7]. We explained these risks to the patient, who despite having a reasonable understanding of the potential harmful consequences of naphthalene use to her, lacked understanding of the full extent of harm to the baby including toxicity and withdrawal. This lack of knowledge had played a significant role in maintaining naphthalene use during the subsequent pregnancy.

She had heard numerous cultural anecdotes about unusual cravings occurring in pregnancies, and she did not see anything amiss about her craving for naphthalene. Having a healthy baby in her second pregnancy despite using naphthalene and not facing any health consequences herself had reinforced her use during this pregnancy. However, when the harmful effects of naphthalene use were pointed out in detail to the patient, she accepted that it was by mere lucky coincidence she or her children did not have to face any harmful effects of naphthalene.

We were not able to explain why the dependence was limited to pregnancy. It could be explained by the neurophysiological changes that occur during pregnancy which promoted initial naphthalene craving in our patient but with repeated inhalation, progressed towards dependence. A study conducted in mice revealed that gestational food craving episodes are associated with a brain connectivity reorganization that affects key components of the dopaminergic mesolimbic circuitry, which drives motivated appetitive behaviours and facilitates the perception of rewarding stimuli. The same study explained that there is a dynamic modulation of dopaminergic signaling through neurons expressing dopamine D2 receptors in the nucleus accumbens, which directly modulate food craving-like events in pregnancy [11]. Interestingly, the neurobiology of substance use also involves the nuceus accumbens and the dopaminergic mesolimbic circuitry.

In conclusion, this was a case of naphthalene dependence confined to pregnancy that resolved spontaneously following delivery, which is rare in the clinical setting and is probably reported for the first time. The case report emphasizes that pregnant women should be screened for psychoactive substance use. Equally important is the need for adequate psychoeducation about the myths and cultural beliefs associated with pregnancy-related cravings and the potentially devastating consequences of various cravings on the neonate and the mother in the event the object of craving is far from being innocuous. The case highlights how chemicals used in day-to-day activities can most unexpectedly lead to potentially life-threatening conditions.

## Data Availability

Not applicable as this is a case report.

## Abbreviations

GABA: Gamma-aminobutyric acid
G.C.E.: General Certificate of Education
NICU: Neonatal Intensive Care Unit
ICD: International Classification of Diseases
NMDA: N-methyl-D-aspartate
